# Medulloblastoma: From TP53 Mutations to Molecular Classification and Liquid Biopsy

**DOI:** 10.3390/biology12020267

**Published:** 2023-02-08

**Authors:** Robert H. Eibl, Markus Schneemann

**Affiliations:** 1 c/o M. Schneemann; Department of Internal Medicine, Hospitals of Schaffhausen, 8208 Schaffhausen, Switzerland; 2 Department of Internal Medicine, Hospitals of Schaffhausen, 8208 Schaffhausen, Switzerland

**Keywords:** medulloblastoma, TP53 mutation, molecular classification, diagnostics, liquid biopsy, animal models, transcriptome, precision oncology

## Abstract

**Simple Summary:**

Medulloblastoma is a common malignant brain tumor in children. Recent progress includes a paradigm shift from histology-based diagnostics to the molecular and genetic profiling of the tumor for an improved correlation with clinical outcomes. This review aims to introduce the clinical physician as well as the basic scientist to a century of research in this field, including the importance of the cell of origin, animal models, and liquid biopsy.

**Abstract:**

A recent paradigm shift in the diagnostics of medulloblastoma allowed the distinction of four major groups defined by genetic data rather than histology. This new molecular classification correlates better with prognosis and will allow for the better clinical management of therapies targeting druggable mutations, but also offer a new combination of monitoring tumor development in real-time and treatment response by sequential liquid biopsy. This review highlights recent developments after a century of milestones in neurosurgery and radio- and chemotherapy, but also controversial theories on the cell of origin, animal models, and the use of liquid biopsy.

## 1. Introduction

A total of 25 years after Eibl detected the first TP53 mutations in tumor probes from medulloblastomas [1], in 2016, the World Health Organization (WHO) introduced a revolutionary paradigm shift in the classification of these and other brain tumors. The molecular profiling of the transcriptome as a new diagnostic system superseded histology (Table 1) [2,3,4]. A recent update in 2021 also included a further analysis of the methylome for epigenetic markers. Over a century after the neurosurgeon Cushing pioneered modern brain tumor surgery [5] and also developed a classification of brain tumors (Table 1) [6], the histologic diagnosis was based on hematoxylin-eosin (HE) stains from formalin-fixed, paraffin-embedded (FFPE) tissue sections and microscopic evaluation by a pathologist, typically detecting a “blue cell” tumor reflecting the high nuclear ratio to the cytoplasm, as well as neuroblastic Homer-Wright rosettes. Immunohistochemistry further improved the diagnostic spectrum through the use of both monoclonal antibodies and polyclonal sera to detect or exclude tumor-related protein markers.

The rationale for molecular classification is a better correlation with biological behavior and to implement new therapies targeting actionable mutations. Although all medulloblastomas are diagnosed as highly malignant grade IV tumors, the four major molecular groups better reflect their development and clinical outcome. Mutations in the TP53 gene indicate a poor outcome when present in one of these four groups but not in another, where they can also be observed less frequently (see Section 2.1). This molecular classification system will be used to guide patients for improved personalized treatment in precision oncology. Some patients should be identified who need a more aggressive radio- and chemotherapy after the surgical removal of the tumor [7]. In contrast, the identification of patients who may not need the most aggressive treatment may benefit from the prevention of dangerous and long-term side effects affecting the developing central nervous system (CNS). Patients may also benefit from combining the new molecular profiling from solid tumor samples with another, also recently emerging milestone in medicine, liquid biopsy, usually from the blood or cerebrospinal fluid (CSF), but also urine which can serve as a low-risk tool to monitor tumor development, as well as providing treatment response significantly earlier than standard medical imaging or CSF cytology. The aim of this review is to provide a timeframe of developments in medulloblastoma diagnostics and treatment, as well as to apply the combination of molecular diagnostics with liquid biopsy in the clinical management of medulloblastoma patients.

**Table 1:** Milestones in medulloblastoma diagnostics, research, and treatment.

Year	Author	Probe	Method	Tumor	Milestone
1910	Wright [8]	Tumor (Autopsy and Biopsy/ Operation)	Histology	Neurocytoma or neuroblastoma (before the creation of the term for medulloblastoma)	A pathologist described CNS tumors differing from most others, later named medulloblastoma. Described as (pseudo-) rosettes, until today referred to as “Homer-Wright” rosettes.
1925	Cushing and Bailey [5]	Neurosurgically removed posterior fossa tumors	Histology	Medulloblastoma	Introduced the term medulloblastoma
1953	Paterson and Farr [9]	Clinical study	Irradiation: 5000 cGy posterior fossa 3500 cGy neuraxis	Reached 65% 3-year survival of medulloblastoma	Irradiation treatment of the whole CNS
1969	Chang et al. [10]	Clinical study	Staging	Medulloblastoma	Staging system
1973	Hart and Earle [11]	Classification	Histology	PNET	Introduced term PNET, regardless of location within CNS
(1950-)1980s	Various authors [12]	Experimental and clinical studies	Development of different chemotherapies and combinations thereof	Brain tumors, incl. medulloblastoma	Introduction of antineoplastic agents for different types of cell cycle, incl. alkylating agents
1991	Eibl and Wiestler [13,14]	Experimentally induced tumors and derived cell lines	Retrovirus-mediated gene transfer of SV40 LT into neural transplants	PNET (indistinguishable from medulloblastoma morphology)	Rat tumor model, histologically identical to human medulloblastoma (neuroblastic rosettes, bipotential differentiation), triggered medulloblastoma research in Bonn and Heidelberg, Germany
1991	Ohgaki, Eibl et al. [1]	Primary tumor tissue	SSCP-PCR, direct sequencing	Medulloblastoma	First detection of p53 mutations in primary medulloblastoma tissue by Eibl, supporting Eibl’s earlier tumor model of the inactivation of p53, also triggered medulloblastoma research, incl. molecular profiling leading to current WHO classification
2001	Reya et al. [15]	Cancer stem cell (CSC)	Compared self-renewal of hematopoetic stem cells with heterogeneity of cancer cells	Migratory cancer stem cells	Established CSC theory (Weissman/Clarke)
2014	Bettegowda et al. [16]	ctDNA	Digital PCR, sequencing	14 tumor types, incl. medulloblastoma	ctDNA detectable for most tumors outside the brain
2016	Louis et al. [2]	Tissue biopsy	Molecular genetics	Medulloblastoma	WHO classification introduced four medulloblastoma groups based on molecular profile (transcriptome)
2018	Garzia et al. [17]	CTC	Parabiotic xenograft model	Medulloblastoma	Hematogenous spread of metastasis to leptomeninges by chemokine-chemokine receptor
2021	Louis et al. [3]	Tissue biopsy	Molecular profile, incl. methylation profile	Medulloblastoma	WHO introduced methylome to further classify medulloblastoma groups
2022	Smith et al. [18]	Normal and tumor tissue	Multi-omics, molecular signatures, expression profiles	Medulloblastoma, groups 3 and 4	Identification of “Cell of origin” in groups 3 and 4 derived from rhombic lip nodulus in developing cerebellum
2022	Hendrikse et al. [19]		Transcriptomics, mutations upstream of CBFA: CBFA2T2, CBFA2T3, PRDM6, UTX, OTX2	Medulloblastoma group 4	Identification of medulloblastoma group 4 progenitor cells in rhombic lip

## 2. Diagnosis—A Century of Debates: Does Medulloblastoma *Per Se* Exist?

Unspecified neurological symptoms, including morning headaches, vomiting, and ataxia, are related to the rapidly growing tumor in the cerebellum or brain stem, mainly in children, which often leads to a blockage of the fourth ventricle, augmenting intracranial pressure. Computed tomography (CT) or magnetic resonance imaging (MRI) can reveal a suspicious mass in the posterior fossa region, which needs to be finally confirmed and graded by a pathologist, or a neuropathologist, to guide the clinicians to various treatment options. The incidence for 0–19 year-old patients is 0.41 per 100,000, but differs with age: 0–4 Years = 0.51; 5–9 Years = 0.63; 10–14 = 0.33; 15–19 Years = 0.16 [20]. Pediatric brain tumors, including the most common medulloblastoma, are the leading cause of tumor-related death in children [21]. Medulloblastoma can regularly form metastasis into the spinal cord, which generally is explained as “drop metastasis” into the ventricle system, followed by transport via the CSF down to the cauda equina, the base of the spinal cord. However, a recent discovery in a parabiotic xenograft model showed an unexpected hematogenous spread of medulloblastoma to the leptomeninges [17]. This was comparable to lymphocyte homing and also involved a chemokine and its receptor [22,23]. Although the metastasis formation of medulloblastoma outside the brain is extremely rare, the cells (”seed”) may frequently enter the bloodstream but do not seem to be competent to survive or grow outside a CNS microenvironment (“soil”). This finding of hematogenous spread supports the application of searching for circulating tumor cells (CTCs) in the blood of medulloblastoma patients (see Liquid Biopsy paragraph). Medical imaging and CSF cytology allow for the detection of common metastasis in the spinal cord independent of the pathway of metastasis (but also without the sensitivity and specificity of liquid biopsy). A pathologist or neuropathologist needs to confirm the diagnosis of the primary tumor in the brain by microscopic analysis (histology). Ideally, the completely new WHO molecular diagnostics (2016, with an update in 2021) is already applied and integrated into the standard histology.

Before the 2021 update of the WHO classification, four major morphological types of medulloblastoma were distinguished by histology: (1) classic, (2) desmoplastic/nodular, (3) medulloblastoma with extensive nodularity (MBEN), and (4) large cell/anaplastic. They are now combined into just one section: “Medulloblastoma, histologically defined”. In contrast, the new and more reliable molecular classification identifies four major groups, as shown in Table 2 [2,3,24].

### 2.1. The New WHO Diagnostic Classification: Activated Oncogenic Signaling Pathways

Two activated signaling pathways have been identified for the first two groups: wingless/Integration-1 (WNT)-activated and sonic hedgehog (SHH)-activated. WNT is a portmanteau for the Drosophila gene “wingless” (Wg), which is detected in mutants lacking wings, and the homologous mouse gene, integration 1 (Int-1), was found earlier to cause tumors by insertional mutagenesis with a retrovirus; SHH refers to the hedgehog gene (hh) found in Drosophila mutants with spikes, reminiscent of a hedgehog (SHH is a vertebrate homolog and named after a character in a video game: Sonic the Hedgehog). It was shown in 2010 [25] that group 1 and 2 medulloblastomas arise from different cells of origin: the WNT group arises outside the cerebellum from the dorsal brainstem and shares most gene activities with lower rhombic lip (LRL) and embryonic brainstem regions, and infiltrates the dorsal brainstem, whereas SHH medulloblastomas are located in the cerebellum. Groups 3 and 4 can also be summarized as non-WNT/non-SHH. Recently, in 2022, the cell of origin for both was identified by multi-omics in the rhombic lip nodulus for cerebellar development in humans [18]. Mutually exclusive mutations in group 4 medulloblastomas could be attributed to the effect of the core binding factor (CBFA) and include *CBFA2T2*, *CBFA2T3*, *PRDM6*, *UTX*, and *OTX2* genes [19]. The expression profiles of group 4 medulloblastoma reflect those of progenitor cells of the subventricular rhombic lip: a specific part of the developing human cerebellum. In contrast to normal cells, which are able to progress from progenitor cells to more differentiated lineages, the tumor cell appears to be stuck at an earlier embryonal stage and produces more, or too many, progenitor-like tumor cells. Although these four groups reproducibly allow prognostic evaluations, with group 1 showing only a low tendency to metastasize and the highest survival rate, it has become clear that there is still heterogeneity within each of the major groups. Therefore, numerous subgroups have been introduced since the WHO classification 2016 (not shown)—and may continue to increase in coming years. Interestingly, 20–30% of the medulloblastomas in the SHH group show TP53 mutations, which confer a poor prognosis. TP53 mutations in the SHH group represent the most important risk factor. In contrast, TP53 mutations can also occur in the WNT group (16%), but then they are not associated with increased risks for a poor outcome and treatment failure [26]. 

Despite the new and independent molecular definition of medulloblastomas, overlapping associations with histology exist, including desmoplastic/nodular medulloblastomas and MBEN, which belong to the SHH-group. Almost all WNT medulloblastomas show classic histology, whereas most large cell/anaplastic medulloblastoma can be found in a specific SHH-subgroup or group 3/group 4-subgroup [2]. The inclusion of distinguishable but biologically different tumors in just one term as medulloblastoma led to the idea that medulloblastoma *per se* does not exist; although these tumors share a common microscopic appearance, they differ in decisive aspects of biological behavior, clinical outcome, and molecular pathways, and therefore need different treatments. Different cells of origin for each group of tumors can explain these discrepancies. Until recently, and due to their identical histological appearance, medulloblastomas were also counted as a member of primitive neuroectodermal tumors (PNETs), a concept that also included neuroblastoma and retinoblastoma. In addition, the identification of SMARCB1-INI1 mutations allows for the diagnosis of some atypical teratoid/rhabdoid tumors (AT/RTs) in the cerebellum, which were formerly considered to be medulloblastomas [27]. Currently, it is accepted that very different tumor entities were formerly summarized as medulloblastoma. Medulloblastomas also arise from different cells of origin and differ from the supratentorial PNETs as well as from AT/RTs.

### 2.2. Cell of Origin

Different lines of evidence—as well as a controversial debate for over a century—have led to the current understanding that medulloblastoma includes independent groups of tumors sharing a basic morphology but not the same cell of origin. Even in 1910, before Bailey and Cushing introduced the term medulloblastoma, pathologist J. Homer Wright separated these tumors composed of primitive neuroepithelial cells from other CNS tumors. In his view, these neurocytomas and neuroblastomas were one entity, which he summarized from autopsies and case reports from the literature, but also neurosurgical biopsies, including one performed by the young neurosurgeon Cushing [8]. Later concepts on the cell of origin remained theoretical or controversial and included classification as sarcomas, neuroblastomas, spongioblastomas (or undifferentiated astrocytic, oligodendral, or ependymal gliomas), or primitive tumors with multidirectional differentiation potential [28]. German pathologists, such as Ribbert, already postulated a different cell of origin for each of these groups of brain tumors, which triggered Bailey for his major brain tumor classification with Cushing. Similar thoughts led to our current nomenclature of tumors, such as astrocytomas, as deriving from astrocytes. On the other hand, one should not imply that any differentiated cell can transform into a tumor cell. As a result of asymmetric cell division, a less differentiated stem cell initiates tumor formation and produces the rare cancer stem cells, but also the more differentiated daughter cells, which further develop into the main tumor mass [15]. With the concept of the cell of origin, and to avoid major confusion with the term spongioblastoma, Cushing and Bailey created the term medulloblast to postulate a hypothetical, embryonic neuroepithelial cell and then introduced medulloblastoma as its own entity different from other CNS tumors. It remains under debate how unique and restricted to the cerebellum such a hypothesized cell needs to be when similar-looking tumors arising from outside the cerebellum, such as neuroblastoma or retinoblastoma. Rubinstein assumed different unique primitive neuroectodermal stem cells in different regions of the CNS, such as retinoblast, glioblast, neuroblast, pineoblast, as well as the medulloblast as the primitive neuroepithelial stem cell in the cerebellum with bipotential, glial or neuronal differentiation potential. Tumors arising from those hypothesized regional stem cells should lead to retinoblastoma, glioblastoma, etc.

### 2.3. Animal Models

**Figure 1:**
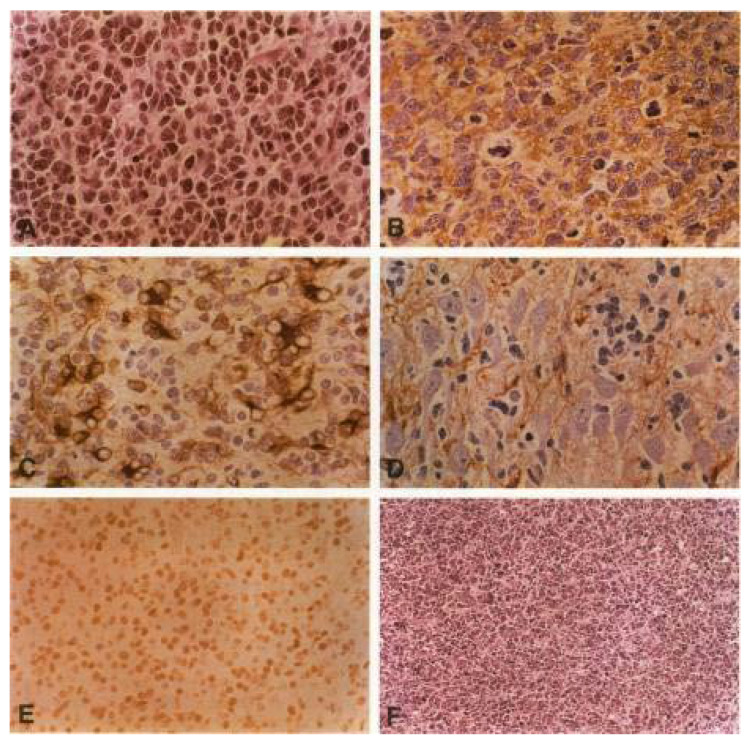
The rat tumor model of PNETs is histologically indistinguishable from human medulloblastoma. Histology of tumors induced in CNS transplants by retroviral gene expression of SV40 large T antigen. (**A**) HE-stain with typical histology, incl. neuroblastic Homer-Wright rosettes as a sign of an early stage neuronal differentiation. (**B**) SYNAPTOPHYSIN expression: immunohistochemical staining with polyclonal antibodies shows a strong synaptophysin expression, indicating neuronal differentiation, (**C**) GFAP expression: astrocytic differentiation of a tumor cell cluster. (**D**) Massive infiltration of tumor cells into the hippocampus of the adjacent host brain. Immunohistochemical staining for synaptophysin. (**E**) Immunohistochemical detection of SV40 large T antigen in the tumor tissue. The monoclonal antibody Pab108 to large T is used for the reaction. Note the characteristic nuclear staining pattern and the absence of immunoreactivity in capillary endothelial cells. (**F**) Secondary transplant obtained after intracerebral injection of a tumor-derived cell line. The typical morphology is completely preserved. (reproduced/adapted from Eibl et al., 1994 [14]).

A number of animal models for PNETs and medulloblastoma-like tumors exist to address the origin and oncogenic pathways of these tumors. In transgenic mouse models, using sequences from DNA viruses such as the SV40 T antigen, tumors were induced in different regions of the CNS, including the pineal gland, depending on the often organ-specific promoter or enhancer sequences used by flanking the transgene [29,30,31,32,33,34]. Eibl and Wiestler introduced a new approach; they transferred the SV40 large T antigen (SV40 LT) into fetal rat brain cells and transplanted them into the brain of adult rats. After long latency periods of 5–11 months, half of the animals developed a typical PNET, histologically indistinguishable from human medulloblastoma [13,14] (Figure 1). This model from Zürich, Switzerland, was reproduced in Bonn and also triggered brain tumor research and neuronal stem cell technology [35], especially at the University of Bonn and the German Cancer Research Center in Heidelberg, Germany. This model triggered the successful search and first detection of TP53 mutations in human medulloblastomas, which finally led to our new understanding of the cell of origin and the new WHO classification of medulloblastoma [35].

**Figure 2:**
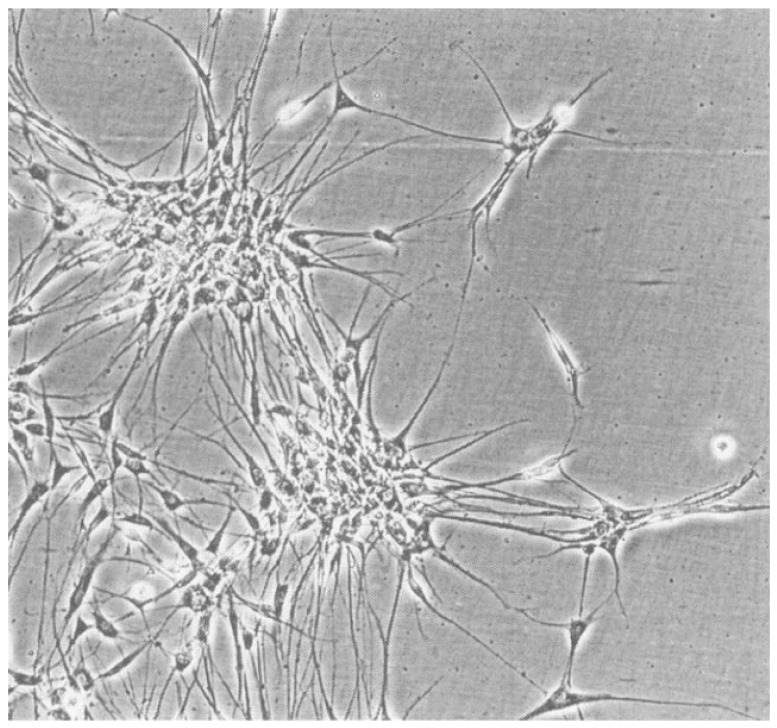
Cell line derived from a SV40 LT-induced PNET. A characteristic property is the formation of cell clusters with long cell processes and other cytological features of primary neuronal cells.

Within a century of controversial debates, some major neuropathologists remained skeptical about how important the search for the cell of origin really was. Rorke [28] challenged the theoretical debates in favor of focusing more on a practical approach to develop better treatments, which can be measured in clinical studies but does not need any hypothetical cell of origin. In fact, even the recent identification of such cells of origin has to prove its clinical value. It was also questioned whether the cell of origin is important for understanding tumor development. Animal models using either the avian DNA virus SV40 or transgenic models using the SV40 large T-antigen (SV40 LT) gene helped to understand both entities better, including primitive neuroectodermal tumors as well as medulloblastomas. Most of the animal models lead to similar medulloblastoma-like tumors in the brain. One exceptional model included a retrovirus-mediated gene transfer of the SV40 LT into transgenic neural transplants in rat brains. After long latency periods of several months, more than half of the animals developed primitive neuroectodermal tumors, morphologically indistinguishable from human medulloblastomas, including a bipotential differentiation potential with neuronal and glial markers, the formation of neuroblastic Homer-Wright rosettes, and a striking migratory potential. The high resemblance to a human tumor is intriguing since many animal tumor models look different from their human counterparts. Cell lines derived from these SV40LT expressing rat medulloblastoma-like tumors showed neuron-like processes (Figure 2) and developed similar tumors after re-transplantation. From immune precipitation studies, it was known that SV40 LT was able to bind to p53 and form complexes [36]. They suggested that the mechanism of action was, therefore, the binding to p53 with the inactivation of a tumor suppressor function, leading to an oncogenic stimulus. Similar models of gene transfer into neural transplants, e.g., Ras, Myc, and RasMyc as a highly oncogenic, cooperating combination of two oncogenes, usually lead to very different tumors in a much shorter time, often in just one or two weeks [37,38,39,40,41]. The beauty of the SV40LT system is the long latency period for tumor development and the suggested additional necessary hit to form these tumors.

### 2.4. First TP53 Mutations

**Figure 3:**
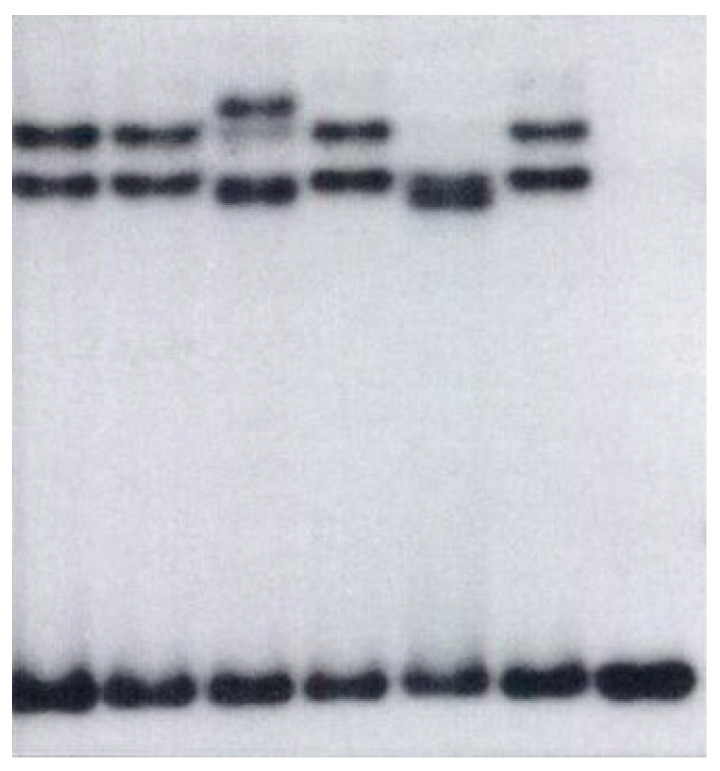
Autoradiography from 1991 of the first detected TP53 mutations in primary medulloblastoma tissue. Extracted DNA from frozen tumor samples was analyzed by the SSCP-PCR of exon 6 of the TP53 gene incorporating radioactively labelled dCTP. Despite the identical length, conformational changes as a result of point mutations in the sequence compared to wild-type DNA can be revealed on an acrylamide gel when run as single-stranded (denatured) DNA fragments. Of five medulloblastomas (lanes 1–5), two (lanes 3 and 5) show a mutation. DNA from the normal brain served as a control, both denatured (lane 6) and normal (double-stranded, lane 7) (reproduced in part from [1], permitted).

SV40 LT was known to bind to TP53, forming complexes, which suggested by functional inactivation as a tumor suppressor. As suggested from his medulloblastoma-like rat tumor model, Eibl then tested a potential inactivation of TP53 by point mutations. With DNA extracts from frozen tumor samples, SSCP-PCR, and direct sequencing of exons, Eibl found the first TP53 mutations in primary medulloblastoma tissue (Figure 3) [1], whereas others were unable to detect such mutations in primary tumor tissue but found one in a cultured cell line, which was assumed to have developed in the culture [42]. TP53 was also detected at that time in other brain tumors of different grades [43]. Eibl detected high frequency even in low-grade astrocytomas, which was fully reproduced with his colleague (von Deimling, Eibl, et al., 1992) [44]. Eibl found no mutations in pilocytic astrocytomas (WHO I) and ependymomas [43]. The Li-Fraumeni syndrome is caused by TP53 mutations in the germline. Those families also developed medulloblastomas despite other tumors. This implies the major function of TP53 in human medulloblastoma development.

## 3. Liquid Biopsy

**Figure 4:**
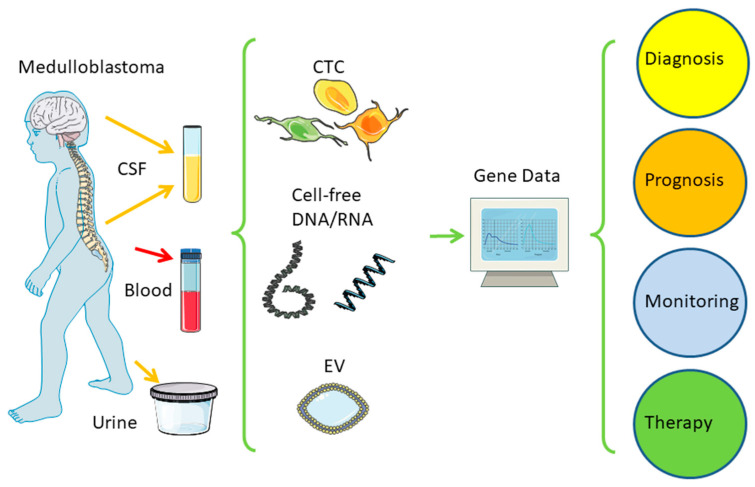
Liquid biopsy. A distance to the tumor in the brain body fluids can be taken at low risk to reveal genetic information from the tumor to guide clinical treatment. CSF—cerebrospinal fluid; CTC—circulating tumor cell; EV—extracellular vesicle. Adapted with permission from [24,46]. Created/modified with SMART [63], licensed by a Creative Commons Attribution 3.0 Unported License [64].

Liquid biopsy has become a milestone in modern medicine [45], which has also been extensively reviewed by Eibl and Schneemann on major cancers [46] and primary brain tumors [47], including medulloblastoma [24] and glioblastoma [48]. A simplified schematic overview is shown in Figure 4. Over the past two decades, different technologies have been developed and applied to detect tumor-derived circulating tumor cells (CTC), cell-free nucleic acids (ctDNA/ctRNA), as well as extracellular vesicles (EV) in body fluids, such as blood, or CSF, but also urine and others. For brain tumors, CSF is currently the best choice when available. Major findings related to the development of liquid biopsy in medulloblastoma are included in Table 1.

**Figure 5:**
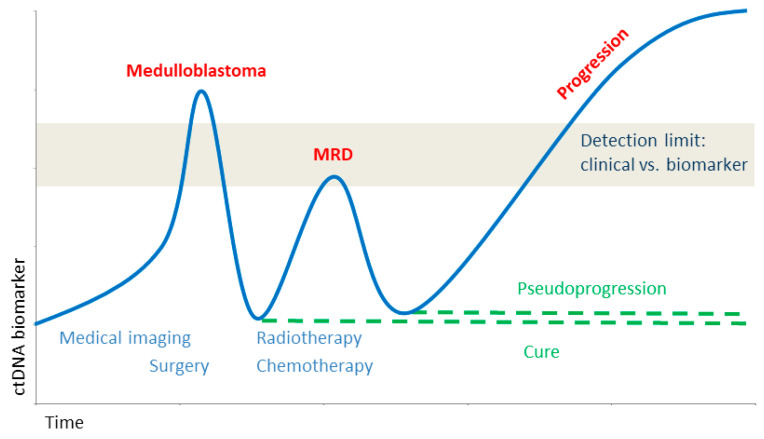
Biomarker during tumor development. Close monitoring of ctDNA allows for the diagnosis as well as early detection of minimal residual disease (MRD) to guide treatment. Adapted with permission from [24,46].

ctDNA from the CSF or blood of medulloblastoma patients is versatile and represents the primary tumor. It can be used to detect MRD or treatment response, including resistance (Figure 5). Recently, only a few clinical studies have started (Table 3, extensively reviewed in [24]), which include the analysis of ctDNA mainly from the blood to confirm the feasibility or to monitor treatment response. This supports the high expectancy of liquid biopsy to enter clinical routines in the future. Currently, a major challenge is a need to standardize procedures to be integrated into clinical routine, which appears to be easier to accomplish for ctDNA. CTCs appear to be the bigger challenge in terms of sensitivity, especially from brain tumors generally lacking the epithelial marker that is used to harvest carcinomas. New approaches need to be further applied and developed, perhaps integrating other markers such as CD44 splice-variants [49], the application of atomic force microscopy (AFM) [50,51,52,53,54,55,56,57,58,59,60], or other biophysical characterization [61]. The challenges for CTC detection need a major research department, equipment, and funding, whereas the ctDNA and mutational analysis appear to be closer to the clinical routines. More and more clinical research groups share their original data to allow for meta-analysis and data mining. For a better comparison, these data should meet the findable, accessible, interoperable, and reusable (FAIR) standards [62].

**Table 3:** Clinical studies using ctDNA or ctRNA from CSF for screening or monitoring medulloblastomas (adapted from [24], with permission).

Year	Author	Tumor	Method	Findings
2020	Escudero et al. [65]	MB	WES, CNVs	ctDNA from CSF sufficient for diagnosis of MB-subgroups, risk stratification, and monitoring (proof of concept study)
2020	Li et al. [66]	Pediatric MB	Whole genome methylation sequencing	High specificity and sensitivity to monitor treatment response of epigenetic signatures in ctDNA from CSF, potential diagnostic, and prognostic value
2021	Liu et al. [67]	MB	WGS	ctDNA from serial CSF samples as prospective marker for MRD in half of patients before radiographic progression
2021	Sun et al. [68]	Pediatric MB	Deep sequencing/NGS, ctDNA in CSF	More alterations detectable in ctDNA from CSF than from primary tumor: a superior monitoring technique when ctDNA is detected from CSF
2022	Lee et al. [69]	MB	RT-PCR sequencing	Circular RNA circ_463 as candidate biomarker
2022	Pagès et al. [70]	Pediatric CNS tumors, incl. MB	ULP-WGS, deep sequencing of specific mutations and fusions	ctDNA is detectable better in CSF than in blood and not in urine. Molecular profiling feasible for small subset of high-grade tumors (incl. MB). Liquid biopsy remains a major challenge for such tumors with low clonal aberrations
2019–2024	NCT03936465 [71] ongoing Phase I study, 66 patients	Pediatric cancer, incl. brain tumors	ctDNA	Clinical toxicity study: ctDNA markers in blood and CSF monitoring treatment response

CNV—copy number variation; CSF—cerebrospinal fluid; MB—medulloblastoma; MRD—minimal residual disease; ULP-WGS—ultra-low-pass whole-genome sequencing; WES—whole exome sequencing.

## 4. Conclusions

A century after medulloblastoma entered the stage as sharing a common neuroepithelial morphology but, distinct from other CNS tumors, genetic and epigenetic analysis have revealed that medulloblastoma *per se* does not exist. Distinct tumor entities have hidden under the umbrella of a hypothetical and transformed medulloblast that was postulated a century ago. The elucidation of mutually exclusive, activated oncogenic signaling pathways also explains the differences in biological behavior and clinical outcome. The recent identification of the cell of origin for medulloblastoma groups 3 and 4 supports the importance of scientific debates for over a century. The rat tumor model for PNETs three decades ago triggered the first detection of TP53 mutations in human medulloblastomas. Interestingly, TP53 mutations in SHH medulloblastoma, but not in WNT, are associated with a poor outcome. After 25 years, these findings have led to the new, genetically defined WHO classification of brain tumors but have also influenced fundamental research on neuronal stem cells [33]. With four distinct groups and several subgroups, the new diagnostic system has clinical relevance and will further be developed for actionable target mutations. The identification of patients with high risks for a poor outcome supports the clinical decision for an aggressive radio- and chemotherapy, whereas the average (or not high) risk patient may be prevented from major side effects with a less aggressive or a later start on a potentially harmful treatment. Liquid biopsy is entering clinical routines and offers the application of current knowledge from transcriptomics and methylomics. New treatment options will have to be developed, including immune or vaccination therapies, to allow new diagnostic achievements to direct the individual patient to the best outcome.

## Figures and Tables

**Table 2:** Molecular classification of medulloblastoma [2,3,24].

Medulloblastoma, Molecularly Defined	Pathway
Group 1	WNT-activated
Group 2	SHH-activated and TP53-wildtype
	SHH-activated and TP53-mutant
Group 3	(non-WNT/non-SHH)
Group 4	(non-WNT/non-SHH)

SHH—sonic hedgehog; WHO—world health organization; WNT—wingless/Integration-1.

## Data Availability

Not applicable.
